# Inhibition of Experimental Choroidal Neovascularization by a Novel Peptide Derived from Calreticulin Anti-Angiogenic Domain

**DOI:** 10.3390/ijms19102993

**Published:** 2018-09-30

**Authors:** Youn-Shen Bee, Yi-Ling Ma, Jinying Chen, Pei-Jhen Tsai, Shwu-Jiuan Sheu, Hsiu-Chen Lin, Hu Huang, Guei-Sheung Liu, Ming-Hong Tai

**Affiliations:** 1 Department of Ophthalmology, Kaohsiung Veterans General Hospital, Kaohsiung 813, Taiwan; ysbee@vghks.gov.tw (Y.-S.B.); pjtasi@vghks.gov.tw (P.-J.T.); sjsheu@vghks.gov.tw (S.-J.S.); sclin@vghks.gov.tw (H.-C.L.); 2 Yuh-Ing Junior College of Health Care & Management, Kaohsiung 807, Taiwan; 3 National Defense Medical Center, Taipei 114, Taiwan; 4 Division of Nephrology, Kaohsiung Veterans General Hospital, Kaohsiung 813, Taiwan; ylma0329@gmail.com; 5 Menzies Institute for Medical Research, University of Tasmania, Hobart, TAS 7000, Australia; janechenjy0907@gmail.com; 6 Department of Ophthalmology, Jinan University, Guangzhou 510632, China; 7 School of Medicine, National Yang-Ming University, Taipei 112, Taiwan; 8 Department of Medical Education and Research, Kaohsiung Veterans General Hospital, Kaohsiung 813, Taiwan; 9 Aier Eye Institute, Aier School of Ophthalmology, Central South University, Changsha 410083, China; huanghu@aierchina.com; 10 Ophthalmology, Department of Surgery, University of Melbourne, East Melbourne, VIC 3002, Australia; 11 Department of Biomedical Sciences, National Sun Yat-Sen University, Kaohsiung 804, Taiwan; 12 Center for Neuroscience, National Sun Yat-Sen University, Kaohsiung 804, Taiwan; 13 Doctoral Degree Program in Marine Biotechnology, National Sun Yat-Sen University, Kaohsiung 804, Taiwan; 14 Graduate Institute of Medicine, Kaohsiung Medical University, Kaohsiung 807, Taiwan

**Keywords:** choroidal neovascularization, neovascular age-related macular degeneration, calreticulin anti-angiogenic domain

## Abstract

Choroidal neovascularization (CNV) is a key pathological feature of several leading causes of vision loss including neovascular age-related macular degeneration. Here, we show that a calreticulin anti-angiogenic domain (CAD)-like peptide 27, CAD27, inhibited in vitro angiogenic activities, including tube formation, migration of endothelial cells, and vascular sprouting from rat aortic ring explants. In a rat model of laser-induced CNV, we demonstrate that intravitreal injection of CAD27 significantly attenuated the formation of CNV lesions as measured via fundus fluorescein angiography and choroid flat-mounts (19.5% and 22.4% reductions at 10 μg and 20 μg of CAD27 injected, respectively). Similarly, the reduction of CNV lesions was observed in rats that had received topical applications of CAD27 (choroid flat-mounts: 17.9% and 32.5% reductions at 10 μg/mL and 20 μg/mL of CAD27 instilled, respectively). Retinal function was unaffected, as measured using electroretinography in both groups receiving interareal injection or topical applications of CAD27 for at least fourteen days. These findings show that CAD27 can be used as a potential therapeutic alternative for targeting CNV in diseases such as neovascular age-related macular degeneration.

## 1. Introduction

Choroidal neovascularization (CNV) is the primary cause of vision loss in patients with wet (exudative or neovascular) age-related macular degeneration (nAMD) and degenerative myopia [1,2]. In these conditions, abnormally high levels of vascular endothelial growth factor (VEGF) are secreted. Elevated levels of VEGF cause the pathological formation of blood vessels in the eye, which can lead to vision loss due to the leakage of blood and fluid into the retina. The recent availability of anti-VEGF therapies (VEGF-neutralizing proteins, such as monoclonal antibodies, antibody fragments, and antibody-receptor fusion proteins) has revolutionized the treatment for CNV by preserving, and even restoring, vision for patients [3,4]. However, existing anti-VEGF therapeutics are expensive and require frequent intravitreal injections (often for many years) to achieve a therapeutic benefit. Moreover, a limited capacity for repeated injections in the public health system poses a barrier to access for patients. Thus, there is an urgency to seek cost-effective, less invasive, and more durable alternative therapies for these conditions.

The calreticulin anti-angiogenic domain (CAD; also known as vasostatin) is the N-terminal domain of calreticulin, comprising amino acids 1–180. It is a potent endogenous inhibitor of angiogenesis [5]. Recombinant CAD has previously inhibited basic fibroblast growth factor (bFGF)- or the VEGF-induced angiogenic response of human endothelial cells [6,7,8] by preventing endothelial cell attachment to laminin, which attenuates the angiogenic response of endothelial cells [9]. CAD has anti-inflammatory properties, which potentiates its anti-angiogenic effects by limiting inflammation-driven angiogenic triggers [10]. Moreover, intramuscular gene delivery or topical application of CAD has been demonstrated to suppress corneal and choroidal neovascularization in rats. Here, we have expanded upon the scope of previous studies by focusing on the functional domain of CAD, which was a cyclic peptide fragment of 27 residues comprising residues 137–163 of calreticulin, referred to as CAD-like peptide 27 (CAD27). Next, we investigated the anti-angiogenic effect and therapeutic efficacy of CAD27 in vitro and in vivo in a rat model of laser-induced CNV via intravitreal administration and topical application.

## 2. Results

### 2.1. Identify the Functional Domain of CAD for the Inhibition of Angiogenesis

**Figure 1:**
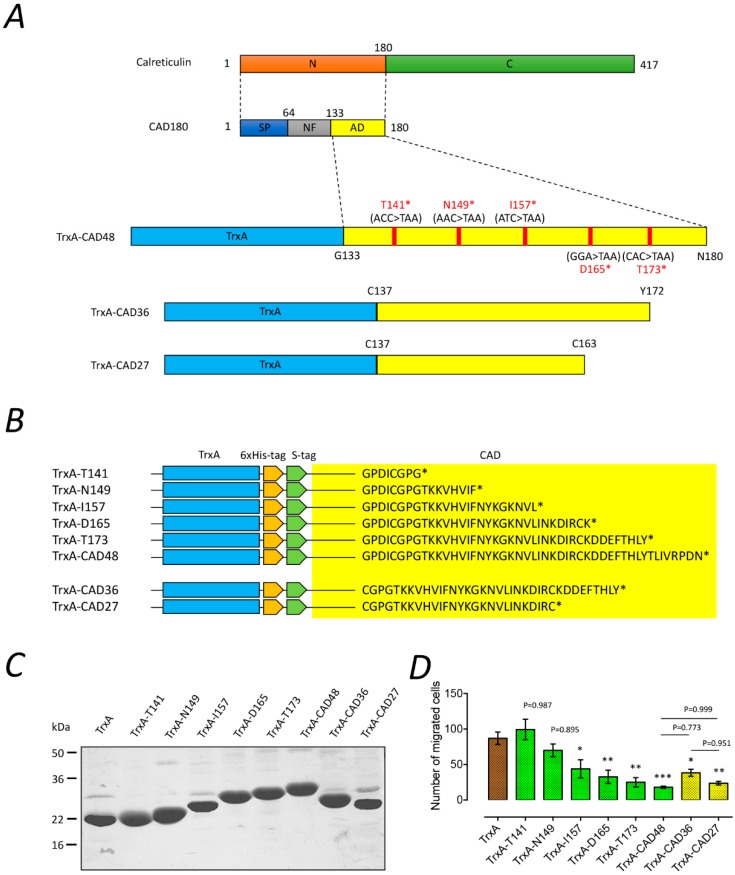
The effect of truncated CAD on the inhibition of angiogenesis. (**A**) Schematic diagram of CAD and its truncated protein constructs. Five truncated CAD fragments derived from CAD48 were generated by introducing a stop codon on each amino acid sequence of T141, N149, I157, D165, and T173. CAD36 (residues C137–Y172) and CAD27 (residues C137–C163) were further generated according to the functional motif of CAD48 and can form in a cyclic structure. N: N-terminal domain. C: C-terminal domain. SP: Signal peptide. NF: Nonfunctional domain. AD: anti-angiogenic domain. (**B**) Amino acid sequence of TrxA-CAD recombinant proteins. The yellow square box indicates truncated CAD fragments. * Stop codon. (**C**) Purification and SDS-PAGE analysis of the recombinant fragments of truncated CAD. (**D**) Effects of the CAD48 and its truncated fragments (green and yellow bars) on migration of primary human endothelial cells (HUVECs). The quantification of migration assay characterizing migrated cells and data are presented as the mean ± SEM (*n* = 3). Statistical analysis between groups was performed using one-way ANOVA followed by Tukey’s multiple comparisons test (* *p* < 0.05, ** *p* < 0.001, *** *p* < 0.0001).

To identify the functional fragment of CAD, a stop codon was introduced into the different amino acid sequences of the thioredoxin (TrxA)-CAD48 construct by site-directed mutagenesis to generate truncated CAD proteins (T141, N149, I157, D165, and T173) (Figure 1A,B). The recombinant proteins were overexpressed as TrxA-tagged proteins in *Escherichia coli* and purified using Ni-NTA affinity chromatography. The purity of the purified proteins was estimated to be approximately 90% (Figure 1C). Subsequently, we performed a Boyden’s chamber migration assay to examine the effects of the truncated CAD fragments on the angiogenic activity of primary human endothelial cells (HUVECs). As shown in Figure 1D, the migration of HUVECs was significantly reduced by the treatment of TrxA-I157, TrxA-D165, and TrxA-T173 protein (TrxA-I157: 44 ± 13, *n* = 3; TrxA-D165: 33 ± 10, *n* = 3; TrxA-T173: 25 ± 7, *n* = 3), whereas the migration of HUVECs was not affected by TrxA, TrxA-T141, and TrxA-T149 protein (the number of migrated cells in TrxA: 87 ± 7, *n* = 3; TrxA-T141: 99 ± 14, *n* = 3; TrxA-T149: 70 ± 9, *n* = 3). These results suggest that the CAD fragments covering 149–165 amino acid residues were responsible for the inhibition of endothelial cell migration. In addition, we further generated two truncated CAD fragments, CAD27 and CAD36, which consisted of residues C137–C162 and C137–Y172 of CAD encompassing the functional motif of the anti-angiogenic domain in a cyclic structure format (Figure 1A,B). Our data showed that TrxA-CAD27 (24 ± 3, *n* = 3) and TrxA-CAD36 (residues C137-Y172; 38 ± 5, *n* = 3) have comparable function in inhibiting the migration of HUVECs to TrxA-CAD48 (18 ± 2, *n* = 3) (Figure 1D).

### 2.2. CAD27 Inhibits the Angiogenic Activity of Endothelial Cells In Vitro and Vascular Sprouting from Rat Aortic Ring Ex Vivo

**Figure 2:**
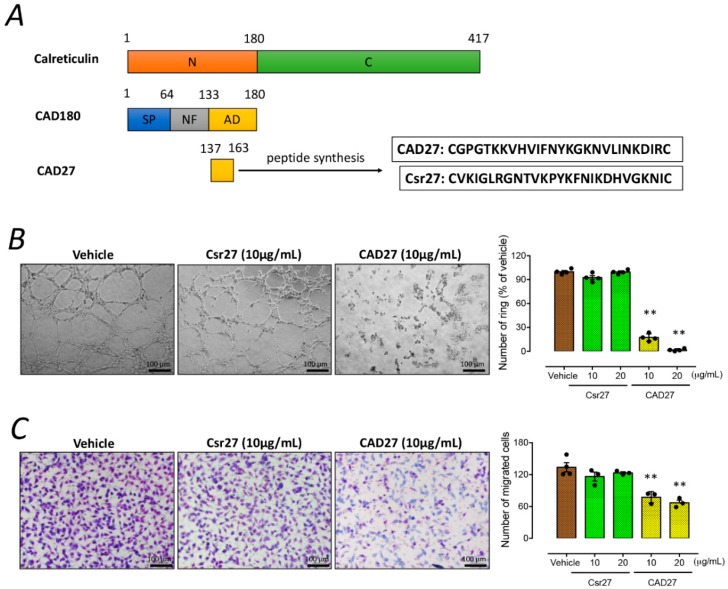
The effect of CAD27 on in vitro angiogenic activities. (**A**) Schematic representation of CAD27. CAD27 was derived from anti-angiogenic domain (residues 137–163) of calreticulin. N: N-terminal domain. C: C-terminal domain. SP: Signal peptide. NF: Nonfunctional domain. AD: anti-angiogenic domain. (**B**,**C**) Effect of CAD27 on tube formation and migration in human endothelial cells (EA.hy926) was assessed. (**B**) Representative images and quantitative analysis of tube formation assay characterizing the lumen formation, and data are presented as the mean ± SEM (*n* = 4). Scale bar: 100 μm. (**C**) Representative images and quantification of migration assay characterizing migrated cells, and data are presented as the mean ± SEM (*n* = 3–4). Scale bar: 100 μm. Statistical analysis between groups was performed using one-way ANOVA followed by Tukey’s multiple comparisons test (** *p* < 0.001).

The CAD27 cyclic peptide was manufactured by de novo synthesis for in vitro and in vivo studies (Figure 2A). To evaluate the anti-angiogenic effect of CAD27 peptides, endothelial tube formation, migration, and rat aortic ring assays were performed. Compared with the vehicle (the percentage of lumen count: 100 ± 1.5%, *n* = 4) or Csr27-treated cells (Csr27 10 μg/mL: 92.6 ± 2.5%, *n* = 4; and Csr27 20 μg/mL: 99.6 ± 1.3%, *n* = 4), cells treated with CAD27 (CAD27 10 μg/mL: 17.4 ± 2.3%, *n* = 4; and CAD27 20 μg/mL: 1.7 ± 0.8%, *n* = 4) showed a significant decrease in their capacity to form tube-like networks on the Matrigel (Figure 2B). Additionally, CAD27-treated cells also showed a poorer migration in the Boyden’s chamber migration assay (the number of migrated cells in vehicle: 134 ± 9, *n* = 4; Csr27 10 μg/mL: 116 ± 8, *n* = 3; or Csr27 20 μg/mL: 123 ± 2, *n* = 3; compared with CAD27 10 μg/mL: 77 ± 6, *n* = 3; or CAD27 20 μg/mL: 67 ± 5, *n* = 3) (Figure 2C).

**Figure 3:**
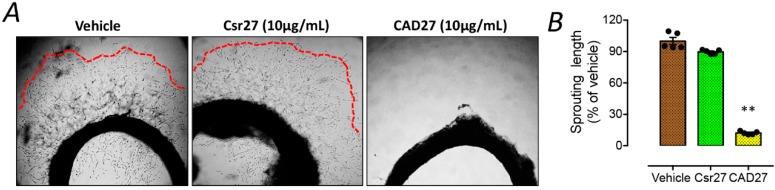
The effect of CAD27 on vascular sprouting from rat aortic ring explants. (**A**) Representative images and (**B**) quantitative analysis of vascular sprouting in 3 week-old rat aortic ring explants. Data are presented as the mean ± SEM (*n* = 5). Statistical analysis between groups was performed using one-way ANOVA followed by Tukey’s multiple comparisons test (** *p* < 0.001). Red lines indicated the border zone of vascular sprouting.

To further validate the anti-angiogenic function of CAD27 ex vivo, the rat aortic rings were embedded in Matrigel to assess microvascular sprouting. A significant reduction of vessel sprouting from aortic ring was found in the CAD27-treated group (the percentage of sprouting length: 12.2 ± 0.7%, *n* = 5) compared to the vehicle-(100 ± 3.4%, *n* = 5) or Csr27-treated group (89.7 ± 0.9%, *n* = 5) (Figure 3).

### 2.3. Effect of Intravitreal or Topical Delivery of CAD27 on Retinal Function in the Rat Retina

Electroretinography (ERG) was employed to evaluate the effects of CAD27 on retinal function in rats 14 days after its intravitreal injection or topical application. There were no statistical differences in the latency and amplitude of the a-wave and b-wave in the eyes which received either the intravitreal injection or topical application of CAD27 compared to those receiving either the vehicle or Lucentis^®^ (ranibizumab) (*n* = 12; Table 1 and Supplementary Figure S1). Thus, our results suggest that intravitreal injection or topical application of CAD27 does not lead to detectably adverse effects on retinal function.

### 2.4. Intravitreal and Topical Delivery of CAD27 Alleviates Laser-Induced CNV Lesions in Rats

**Figure 4:**
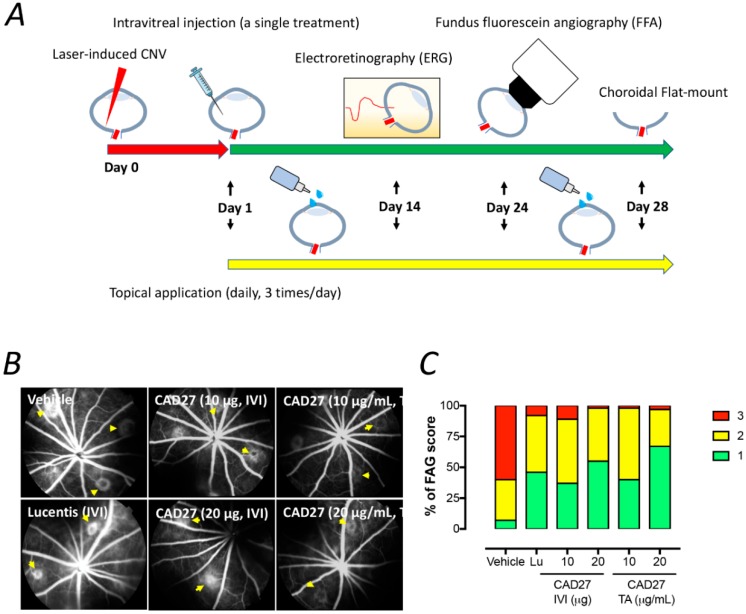
Fluorescein angiographic analysis of choroidal neovascularization (CNV) lesions after an intravitreal or daily topical application of CAD27. (**A**) A schematic diagram of the timeline for the laser-induced CNV rat model, treatments, and examination. Choroidal vascularity of laser-induced CNV lesions was examined by FFA (day 24) and choroidal flat-mount labeling with FITC-dextran (day 28) after a single intravitreal injection or daily topical application of CAD27. (**B**) Representative CNV lesions in rat eyes were identified by fundus fluorescein angiography after an intravitreal or daily topical application of CAD27. Yellow arrows indicated the lesions of CNV. (**C**) CNV lesions from fluorescein angiography were analyzed at days 24 after treatment, and data are presented as percentage of CNV score (*n* = 26–46 from 6 to 8 eyes). Score 1 (green) indicates staining, Score 2 (yellow) indicates moderate leakage, and Score 3 (red) indicates heavy leakage. Lu: Lucentis^®^, IVI: intravitreal injection, TA: topical application.

A rat model of laser-induced CNV was employed to evaluate the therapeutic potential of intravitreal and topical delivery of CAD27. One day after the laser surgery, CAD27 was administered via a single intravitreal injection or daily topical application (three times a day) in the CNV rats. The extent of choroidal vascularization was examined using fundus fluorescein angiography and choroidal flat-mounts with FITC-dextran perfusion on days 24 and 28 after CNV induction (Figure 4A), respectively. Compared to the vehicle-treated eyes (60% of the eyes had score 3, 33% had score 2, and 7% had score 1, *n* = 42), intravitreal injection of CAD27 (10 μg CAD27: 11% of the eyes had score 3, 52% had score 2, and 37% had score 1, *n* = 27; 20 μg CAD27: 2% of the eyes had score 3, 43% had score 2, and 55% had score 1, *n* = 40) and Lucentis^®^ (8% of the eyes had score 3, 46% had score 2, and 46% had score 1, *n* = 26) reduced the CNV score, measured by FFA, on day 24 (Figure 4B,C). Similarly, the daily topical application of CAD27 also reduced the CNV score (10 μg/mL CAD27: 2% of the eyes had score 3, 58% had score 2, and 40% had score 1, *n* = 46; 20 μg/mL CAD27: 3% of the eyes had score 3, 30% had score 2, and 67% had score 1, *n* = 44) (Figure 4B,C).

**Figure 5:**
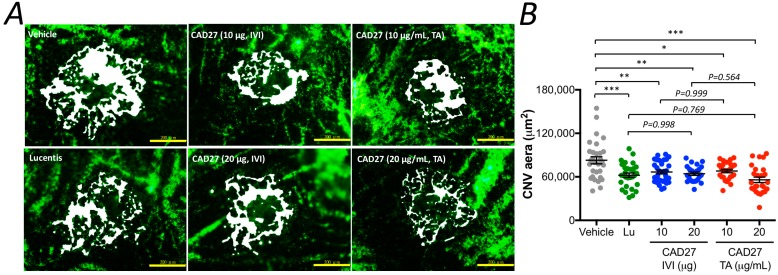
Flat-mount analysis of choroidal vascularity after an intravitreal or daily topical application of CAD27. Choroidal vascularity of laser-induced CNV lesions was examined by labeling using FITC-dextran. (**A**) Representative profile of FITC-dextran-positive blood vessels in choroidal flat-mounts at day 28 after treatment. Scale bar: 200 μm. (**B**) FITC-dextran labeling CNV in the choroidal flat-mounts was quantified and data are presented as mean ± SEM (*n* = 26–31 from 6 to 8 eyes) using one-way ANOVA followed by Tukey’s multiple comparisons test (* *p* < 0.05, ** *p* < 0.001, *** *p* < 0.0001). White area indicated the lesions of CNV in choroid flat-mount. Lu: Lucentis^®^, IVI: intravitreal injection, TA: topical application.

To further confirm the therapeutic potential of CAD27, the size of the CNV lesion was measured using the flat-mount analysis after perfusion with FITC-dextran on day 28 (Figure 5A and Supplementary Figure S2). Compared to the vehicle-treated eyes (the CNV size: 82,867 ± 4880 (95% CI: 73,303–92,430) μm^2^, *n* = 30), a significant reduction in the size of CNV lesion was found in the rat eyes that had received an intravitreal administration of CAD27 (CAD27 10 μg: 66,714 ± 2589 (95% CI: 61,640–71,787, *n* = 31) μm^2^ and CAD27 20 μg: 64,327 ± 2341 (95% CI: 59,738–68,915, *n* = 23) μm^2^) and Lucentis^®^ (62,233 ± 3050 (95% CI: 56,256–68,209, *n* = 30) μm^2^) as well as daily topical application of CAD27 (CAD27 10 μg/mL: 67,959 ± 2313 (95% CI: 63,425–72,492, *n* = 26) μm^2^ and CAD27 20 μg/mL: 55,911 ± 3771 (95% CI: 48,519–63,302, *n* = 26) μm^2^; Figure 5B). These results indicate that intravitreal and topical application of CAD27 attenuated the severity of experimental CNV.

## 3. Discussion

In the present study, we have identified the core anti-angiogenic domain of CAD and demonstrated that the de novo synthetic CAD27 cyclic peptides can inhibit angiogenesis in vitro and ex vivo and suppress ocular neovascularization in vivo. Specifically, we have confirmed the anti-angiogenic activity of CAD27 by its inhibition of endothelial tube formation and migration, as well as its capacity to reduce the incidence of vascular sprouting from rat aortic ring. Intravitreal and topical application of CAD27 attenuated laser-induced CNV in rats as revealed by using FFA and choroidal flat-mount, and no detectable adverse effects on retinal function was found by ERG.

Treatment for nAMD has recently been revolutionized by the availability of intravitreal anti-VEGF agents [11]. Such agents that bind to VEGF, thereby preventing Flt-1 and KDR/Flk-1 signaling and inhibiting the neovascular response, have been shown to be superior to previous treatment modalities such as verteporfin photodynamic therapy [12]. While there is promise for improvement in vision with intravitreal anti-VEGF agents, there are also shortcomings in terms of variable response to therapy as well as loss of efficacy in a subgroup of patients. Over the years, many endogenous inhibitors of angiogenesis including various anti-angiogenic peptides, hormone metabolites, and apoptosis modulators have been discovered and proposed as potential therapeutic alternatives for targeting neovascularization and/or excessive vascular leakage in the eye [13]. Some of them have reached clinical trial stages including Pigment epithelium-derived factor (ClinicalTrials.gov Identifier: NCT00109499) and endostatin/angiostatin (RetinoStat) (ClinicalTrials.gov Identifier: NCT01678872 and NCT01301443) for the treatment of nAMD. Similar to these endogenous angiogenesis inhibitors, CAD is also a naturally occurring anti-angiogenic peptide derived from human calreticulin. Unlike most of the angiogenesis inhibitors, which appear to have more complex activities, CAD specifically targets proliferating endothelial cells with low toxicity [7,14,15]. Moreover, CAD appears to have a four- to ten-fold lower effective dose then endostatin and angiostatin for angiogenesis inhibition in vivo [16,17], as well as having anti-inflammatory properties that will help to control inflammation, which is a major contributor to the ongoing drive for neovascularization in nAMD [10]. These favorable features make CAD superior to previously identified angiogenesis inhibitors, which are derived from fragments of endogenous precursor proteins, and current therapeutic approaches (e.g., anti-VEGF antibody injections).

Our previous studies have demonstrated that the gene delivery of CAD and its derived fragment (CAD112) can attenuate the development of choroidal and retinal neovascularization in rodent models of laser-induced CNV and oxygen-induced retinopathy [18]. However, adverse effects associated with prolonged expression of vascular targeting proteins by gene delivery may have unwanted side effects, including retinal vascular toxicity. There are also other major obstacles to the acceleration of gene therapy from bench to bedside, such as cost of the treatment [19]. Therefore, in the present study, we rationally designed a small fragment of CAD, CAD27, which covers the core anti-angiogenic domain of CAD and can be produced by chemical synthesis at a lower cost than its parent molecule, recombinant CAD protein or CAD gene therapy. Moreover, the CAD27 peptide can be cyclized by head-to-tail to form a secondary cyclic structure, which is linked by a disulfide bond between cysteine residues through cysteine 1–27. The cyclic engineering peptide presents several additional properties, such as its large surface area-to-volume ratio conferring high affinity and selectivity for the target ligand, high stability, and lower immunogenicity and low toxicity, offering a promising approach to improve its biological activity [20,21]. Our data show that synthetic CAD27 peptide treatment provided similar benefits in the inhibition of CNV formation compared to recombinant CAD protein (CAD112), suggesting that the chemically synthetic peptides did not alter its anti-angiogenic properties (Supplementary Figure S3). In addition, intravitreal and topical application of CAD27 also showed a similar inhibitory effect on the reduction of CNV lesions compared with Lucentis^®^ (ranibizumab), a standard treatment option for nAMD. Thus, these data make a compelling case that CAD27 delivered by intravitreal or topical application can be used as a therapeutic alternative to conventional therapies for pathologic ocular neovascularization.

Intravitreal and topical routes were used to assess the therapeutic effect of CAD27 for the treatment of CNV. Intravitreal injection has been considered an effective way to administer pharmacological treatments to the eye for managing pathological conditions associated with abnormal blood vessel growth, such as nAMD and diabetic retinopathy, which require life-long, frequent intravitreal injections. Nevertheless, retinal specialists are not easily accessible in either regional communities, or less developed or developing countries for intraocular injections, meaning that the diseases will eventually progress to blindness. Intravitreal injection also carries risks of potentially blinding complications and serious intraocular infections. The topical application of ophthalmic formulation is the most convenient, safe, effective, and least invasive drug delivery method, and could potentially eliminate the risks associated with eye injections, as well as increase accessibility for patients. Several studies have previously demonstrated the feasibility of topical application of ophthalmic formulation for the management of CNV [22,23,24,25]. Therefore, in the present study, we have assessed the therapeutic potential of CAD27 for targeting CNV delivered via both intravitreal injection and topical application. Indeed, our data indicate both delivery routes provide similar benefits in reducing CNV lesions, suggesting that both the drug delivery methods are available and effectual in the rat laser-induced CNV model. These data are also consistent with our previous study which showed that the topical delivery of recombinant CAD proteins (CAD180 and 48) attenuates the development of CNV in the laser-induced CNV model [26,27]. Moreover, CAD27 may have additional benefits over CAD180 or 48 as it can be chemically synthesized and possesses a low molecular weight which allows it to have better retinal or transscleral penetration to the posterior segment when administered through intravitreal injection and topical application, respectively. Further research is needed to confirm its pharmacokinetic profile and bioavailability in the eye, particularly following topical administration. In addition, by using in vivo ERG assessment, we showed that intravitreal or topical application of CAD27 had little effect on retinal function over the course of 14 days. However, the long-term safety of CAD27-based therapy will need to be confirmed before their translation into clinical trials.

In summary, our study demonstrates that the therapeutic delivery of CAD27 attenuates the formation of CNV in vivo. Although further investigations are required to assess its pharmacokinetic profile and long-term efficacy, our data suggest that the topical application of CAD27 may be a viable therapeutic alternative for CNV, as it does not require ocular injection and can thus circumvent the risks associated with frequent injections required for current therapies.

## 4. Materials and Methods

### 4.1. Site-Directed Mutagenesis

CAD48 cDNA was amplified via polymerase chain reaction (PCR) from CAD and subcloned into the restriction digest sites of NdeI and XhoI of the pET32a(+) vector (catalog no. 69015, Novagen Inc., Madison, WI, USA) to yield the pET32a(+)-CAD48 plasmid (Supplementary information). Point mutations were introduced by PCR using the QuikChange Site-Directed Mutagenesis kit (catalog no. 200519, Agilent Technologies, Santa Clara, CA, USA) according to the manufacturer’s instructions. The mutagenic oligonucleotide primers were designed using a web-based QuikChange Primer Design Program (www.agilent.com/genomics/qcpd) and are shown in Supplementary Table S1. PCRs for single amino acid mutations were run for 18 cycles of 30 s at 95 °C, 1 min at 55 °C, followed by 1 min at 68 °C. The resulting mutant plasmids were verified by DNA sequencing.

### 4.2. Expression and Purification of Recombinant TrxA-Tagged Truncated CAD Protein

Recombinant TrxA-CAD was expressed and purified as previously described [7]. Briefly, pET32a(+)-CAD48 or mutant plasmid was transformed in BL21(DE3)pLysS Competent Cells (catalog no. 69451, Novagen Inc.) and the transformed cells were grown at 37 °C until log-phase (optical density (OD 600 nm) of 0.5 to 0.9). Expression of P protein was induced by the addition of 1 mM isopropyl thiogalactose (IPTG; catalog no. I6758, Sigma-Aldrich, St. Louis, MO, USA) and the culture was incubated for an additional 3 h. The cell pellet was harvested by centrifugation at 5000 rpm for 10 min at 4 °C, resuspended in the binding buffer (20 mM phosphate buffer, pH 7.4, 20 mM imidazole, 150 mM NaCl, 1 mM EDTA, 1 mM PMSF, 1 μg/mL aprotinin, 1 μg/mL leupeptin, and 1 μg/mL pepstatin), and then homogenized by sonication. After centrifugation at 12,000 rpm for 20 min at 4 °C, the supernatant was mixed with Ni-NTA agarose (catalog no. 30210, Qiagen, Valencia, CA, USA) at 4 °C for 30 min. The beads were washed four times with the binding buffer, and the recombinant protein was eluted with buffer (20 mM phosphate buffer, pH 7.4, 250 mM imidazole, 150 mM NaCl). Salted and endotoxin were removed by passing through a G-25 Sephadex column (catalog no. 17085101, GE Healthcare Life Sciences, Pittsburgh, PA, USA) and Detoxi-G gel (catalog no. 88270, Pierce, Rockford, IL, USA).

### 4.3. Preparation of CAD27 Peptide

CAD27 peptide (CGPGTKKVHVIFNYKGKNVLINKDIRC) and the presumed nonfunctional form of the scrambled peptide (Csr27; CVKIGLRGNTVKPYKFNIKDHVGKNIC) were manufactured by de novo peptide synthesis (Kelowan Incs, Taipei, Taiwan). The synthesized peptides were reconstituted in Dulbecco’s Phosphate Buffered Saline (DPBS; catalog no. 14190144, Gibco™, Invitrogen, Carlsbad, CA, USA) for in vitro and in vivo studies.

### 4.4. Cell Culture

Human primary umbilical vein endothelial cell, HUVEC, was purchased from Lonza (catalog no. CC-2519; Walkersville, MD, USA) and cultured in endothelial cell basal medium-2 (EBM-2) supplemented with the EGM™-2 BulletKit™ (catalog no. CC-5035; Lonza) in a humidified 5% CO_2_ incubator at 37 °C. Human endothelial cells line, EA.hy926, was purchased from ATCC (CRL-2922™) and cultured in Dulbecco’s Modified Eagle’s Medium (DMEM; catalog no. 11965118, Invitrogen) supplemented with Penicillin-Streptomycin (50 U/mL; catalog no. 15140122, Invitrogen), 10% fetal bovine serum (FBS; Gibco™, Invitrogen) and l-glutamine (2 mM; catalog no. 25030081, Invitrogen) in a humidified 5% CO_2_ incubator at 37 °C.

### 4.5. Tube Formation Assay

Quantification of tube formation was performed using a previously described method [26]. Briefly, a 24-well plate was pre-incubated with BD Matrigel™ Basement Membrane Matrix (catalog no. 356234, BD Biosciences, Franklin Lakes, NJ, USA) at 37 °C for 30 min. Cells were incubated with DPBS, CAD27 (10 and 20 μg/mL), or Csr27 (10 and 20 μg/mL) at 37 °C for 5 h. Cells (1.5 × 10^4^) were resuspended in the completed medium and loaded on the top of the Matrigel. Following 6 h incubation at 37 °C, each well was photographed under a bright field phase contrast microscope. The number of endothelial tube lumens was counted in three replicate wells and only the completed ring structures created by three to five endothelial cells were considered as tubes. The analysis was performed in Image J version 1.48 software (http://imagej.nih.gov/ij/; provided in the National Institutes of Health, Bethesda, MD, USA).

### 4.6. Cell Migration Assay

Cell migration was performed in a Boyden’s chambers (catalog no. CBA-100-C, Cell Biolabs, INC, CA, USA), which comprises upper and lower systems separated by a 0.005% gelatin coating 8-µm pore size polycarbonate membrane, as previously described [26]. Cells (2 × 10^4^) were resuspended in serum-free medium, loaded onto the upper well and incubated with vehicle (DPBS), CAD27 (10 and 20 μg/mL), or Csr27 (10 and 20 μg/mL), respectively, in a humidified 5% CO_2_ incubator at 37 °C for 6 h. The cells on the upper side of the filter were removed. Those that had migrated to the lower side were fixed in absolute methanol, stained with 10% Giemsa solution (Merck, Darmstadt, Germany) and five high power fields from each well were counted under a bright field phase contrast microscope (Olympus BX40; Olympus Optical Co., Tokyo, Japan).

### 4.7. Aortic Ring Assay

This ex vivo aortic ring angiogenesis assay was performed as described previously [26]. Briefly, the thoracic aortas were excised from 3 week-old male Sprague Dawley rats and immediately placed into prechilled DMEM containing 10% FBS. Clotted blood inside the aorta was flushed with media, and the peri-adventitial fibroadipose tissue was removed. Aortas were then cut into cross-sectional rings approximately 1–1.5 mm in length. Rings were placed into a 24-well plate containing 0.5 mL of cold BD Matrigel™ Basement Membrane Matrix supplemented with MCDB131 medium (catalog no. 10372019, Invitrogen) and incubated at 37 °C until the Matrigel polymerized. Subsequently, aortic rings were treated with vehicle (DPBS), CAD27 (10 μg/mL), or Csr27 (10 μg/mL) and maintained in a humidified 5% CO_2_ incubator at 37 °C for five days. Microvascular sprouting from each aortic ring were examined and imaged daily under a bright field phase contrast microscope (Olympus BX40, Shinjuku, Tokyo, Japan). The greatest distances from the aortic ring body to the end of the vascular sprouts (sprout length) were measured by Image J version 1.48 software at three distinct points per ring and in three different rings per treatment group.

### 4.8. Animal and Ethical Approval

All animals were handled in accordance with the ARVO Statement for the Use of Animals in Ophthalmic and Vision Research for the experiments performed in this study, and was obtained from the Institutional Animal Care and Use Committee (IACUC) of Kaohsiung Veterans General Hospital (vghks-103-A010, 26 July 2013). The pigmented Brown Norway rats (eight week-old, female) and Sprague Dawley rats (three week-old, male) were purchased from National Animal Center, Taipei, Taiwan. Rats were housed in standard cages, with free access to food and water in a temperature-controlled environment under a 12-h light (50 lux illumination) and 12-h dark (<10 lux illumination) cycle to minimize possible light-induced damage to the eye.

### 4.9. Generation of CNV by Laser Photocoagulation

The CNV lesions were induced in rat eyes by laser photocoagulation as previously described [26]. Briefly, Brown Norway rats were anesthetized with an intramuscular injection of a mixture of 2% xylocaine (0.15 mL/kg body weight, Astra, Astra Sodertalje, Sweden) and ketamine (50 mg/kg body weight, Parke-Davis, Morris Plains, NJ, USA). Pupils were dilated with 1% tropicamide (Alcon Laboratories, Fort Worth, TX, USA). A piece of cover glass served as the contact lens to improve the visibility of the fundus. Argon laser (Novus Omni; Coherent, Palo Alto, CA, USA) irradiation was delivered through a slit lamp (Carl Zeiss, Oberkochen, Germany). Laser parameters: spot size of 50 µm, power of 400 mW, and exposure duration of 0.05 s. Disruption of Bruch’s membrane was detected by the emergence of a bubble at the center of photocoagulation in the laser spotted zone. Six lesions were generated in each eye at the 1, 3, 5, 7, 9, and 11 o’clock positions located at equidistance from the optic disk and between the major retinal vessels.

### 4.10. Intravitreal Injection and Topical Application

One day after laser-induced CNV induction, rats were anesthetized with a combination of xylocaine (0.15 mL/kg body weight) and ketamine (50 mg/kg body weight). Intravitreal injection was performed under a surgical microscope as previously described [18]. After a small puncture through the conjunctiva and sclera was created using a 30 gauge needle, a 32 gauge blunt needle connected to a 10-μL Hamilton syringe was inserted into the vitreous and 5 μL of DPBS suspension containing Lucentis^®^ (ranibizumab, 50 μg; Novartis, Basel, Switzerland), CAD27 (10 and 20 μg), or vehicle (DPBS) was injected into one eye of each rat using a UMP3–2 Ultra Micro Pump (World Precision Instruments, Sarasota, FL, USA) at a rate of 100 nL/s. Only a single injection was permitted in the CNV rat. The CAD27 (10 and 20 μg/mL) were formulated as eye drop in DPBS and topical installed three times a day in the rat eye after CNV induction for 28 days.

### 4.11. Electroretinogram (ERG)

The single bright flash ERGs (UTAS-E 300; LKC Technology, Gaithersburg, MD, USA) under a dark-adapted environment (~12 h) were performed to assess the effect of intravitreal or topical administration of CAD27 on retinal function. After at least 30 min of darkness adaptation, rats were anesthetized. Gold foil was placed on the cornea with 2% methylcellulose gel (Omni Vision, Neuhausen, Switzerland). A reference electrode was attached to the shaven skin of the head and a ground electrode clipped to the rat’s ear. After reducing the background noise below 60 Hz, a single flash of bright light (duration, 100 ms), 30 cm from the eye, was used as the light stimulus. Responses were amplified with a gain setting ±500 μV and filtered with low 0.3 Hz and high 500 Hz from an amplifier. Data were acquired, digitized, and analyzed using EM for Windows, version 2.6.

### 4.12. Fundus Fluorescein Angiography (FFA)

The size of CNV lesions were evaluated by FFA analysis using a digital fundus camera (Visupac 450, Ziess FF450, Oberkochen, Germany) on day 24 after laser photocoagulation. The rats were anaesthetized and the fluorescein sodium solution (10% Fluorescite; Alcon, Fort Worth, TX, USA) was intraperitoneally injected at a dose of 0.1 mL/kg body weight. Late-phase angiograms were obtained at 8 min after injection, and digital fundus pictures of bilateral eyes were taken within 1 min. The incidence of choroidal neovascularization was defined when early hyperfluorescence with the onset of late leakage was present at the site of laser injury [26]. The extent of the leakage of the CNV lesions were graded using a leakage score system. Score 0 indicates no staining (no hyperfluorescence), Score 1 indicates staining (hyperfluorescence without leakage), Score 2 indicates moderate leakage (hyperfluorescence in the early or midtransit images and late leakage), and Score 3 indicates heavy leakage (bright hyperfluorescence in the transit images and late leakage beyond treated areas). The scores were assessed by two independent ophthalmologists who were masked to the experimental design.

### 4.13. Quantification of Choroidal Vascularity by Flat-Mounted Analysis

Rats were euthanized 28 days after laser photocoagulation. The choroidal blood vessels in rat eyes were labeled by perfusion with fluorescein isothiocyanate (FITC)-dextran (2 × 10^6^ MW; catalog no. FD2000S, Sigma-Aldrich, St. Louis, MO, USA) [28,29]. Briefly, the rats were anaesthetized and subjected to an intracardiac perfusion of approximately 50 mL of lactated Ringer solution, followed by 20 mL of FITC-dextran in lactated Ringer solution (5 mg/mL) with gelatin (10%, *w*/*v*; catalog no. G9382, Sigma-Aldrich, Saint Louis, MO, USA). The eyes were enucleated and fixed in 10% phosphate-buffered formalin for 2 h at room temperature. After the cornea and lens were removed, the retinal pigment epithelium (RPE)/choroid/sclera flat-mounts were obtained on microscopic slides. Flat-mounts were imaged with a laser-scanning confocal fluorescence microscope. The areas of hyperfluorescence associated with each CNV lesion was measured by observers who were blinded to the groups using ImageJ version 1.48 software.

### 4.14. Statistical Analysis

Results are presented as means ± standard errors of the means (SEM). The experimental data was analyzed with one-way ANOVA followed by Tukey’s multiple comparisons test or two-tailed Student’s *t*-test (GraphPad Prism software version 7.0). A value of *p* less than 0.05 was considered statistically significant.

## Figures and Tables

**Table 1:** The effect of intravitreal and topical application of CAD27 on retinal function assessed by electroretinography (ERG).

ERG Parameters	Vehicle	Lucentis^®^ (IVI)	CAD27 (10 μg, IVI)	CAD27 (20 μg, IVI)	CAD27 (10 μg/mL, TA)	CAD27 (20 μg/mL, TA)	*p* Value
a-wave amplitude, μV	−128.6 ± 11.1	−145.7 ± 10.9	−161.4 ± 14.7	−142.1 ± 10.2	−159.9 ± 10.3	−163.3 ± 9.2	0.1557
a-wave latency, ms	19.7 ± 0.7	20.0 ± 0.6	17.8 ± 0.9	19.8 ± 1.0	18.2 ± 0.5	18.7 ± 0.4	0.0834
b-wave amplitude μV	296.5 ± 15.2	314.3 ± 20.8	351.1 ± 27.2	310.1 ± 29.7	336.5 ± 19.9	367.1 ± 22.4	0.1522
b-wave latency, ms	58.7 ± 1.5	55.1 ± 1.4	51.9 ± 2.2	52.2 ± 2.5	54.8 ± 1.5	54.2 ± 1.3	0.0675

Statistical analysis was performed using one-way ANOVA. IVI: intravitreal injection, TA: topical application.

## Supplementary Materials

Supplementary materials can be found at http://www.mdpi.com/1422-0067/19/10/2993/s1.
