# Supplementary material for: Inhibition of Experimental Choroidal Neovascularization by a Novel Peptide Derived from Calreticulin Anti-Angiogenic Domain
Source: Int J Mol Sci. 2018 Sep 30;19(10):2993. doi: 10.3390/ijms19102993 (PMC6213176; doi:10.3390/ijms19102993)
Supplement: Supplementary file 1 [file ijms-19-02993-s001.pdf]

Supplementary Table S1. Mutagenic primers for site-directed mutagenesis.

Primer	Sequence
T173 FWD	5'-GAGTTTACACACCTGTACTAACTGATTGTGCGGCCAGAC-3'
T173 REV	5'-GTCTGGCCGCACAATCAGTTAGTACAGGTGTGTAAACTC-3'
D165 FWD	5'-CAAGGACATCCGTTGCAAGTAAGATGAGTTTACACACCTGT-3'
D165 REV	5'-ACAGGTGTGTAAACTCATCTTACTTGCAACGGATGTCCTTG-3'
I157 FWD	5'-GGCAAGAACGTGCTGTAAAACAAGGACATCCGTTGCAAGGATGATG-3'
I157 REV	5'-CATCATCCTTGCAACGGATGTCCTTGTTTTACAGCACGTTCTTGCC-3'
N149 FWD	5'-GAAGGTTCATGTCATCTTCTAATACAAGGGCAAGAACGTGC-3'
N149 REV	5'-GCACGTTCTTGCCCTTGTATTAGAAGATGACATGAACCTTC-3'
T141 FWD	5'-ATCTGTGGCCCTGGCTAAAAGAAGGTTCATGTCATCTTCAACTACAA-3'
T141 REV	5'-TTGTAGTTGAAGATGACATGAACCTTCTTTTAGCCAGGGCCACAGAT-3'

FWD: forward primer. REV: Reverse primer.

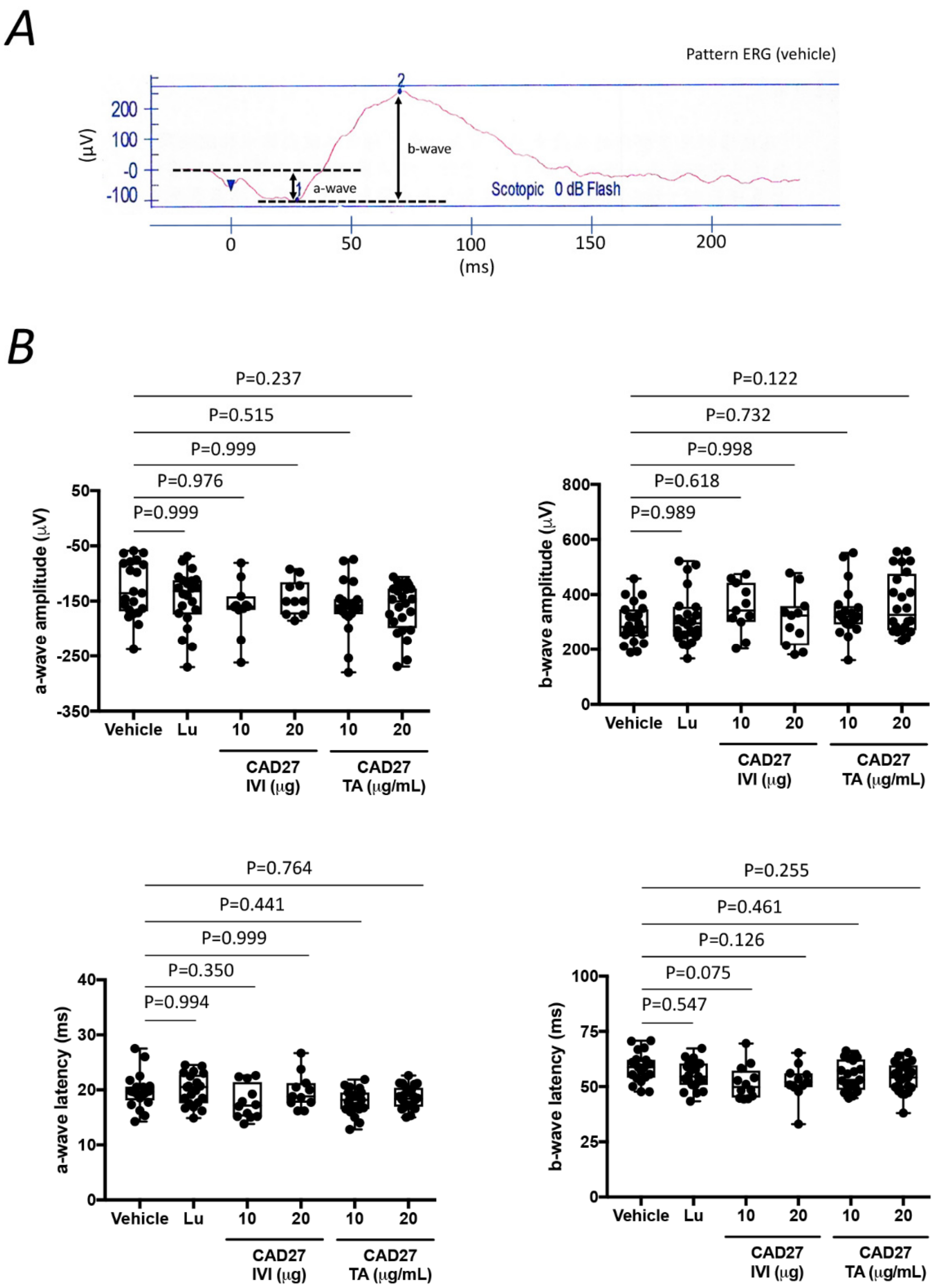

Supplementary Figure S1. The effect of intravitreal and topical application of CAD27 on retinal function. (**A**) Schematic representation of ERG waveforms at selected intensities from control (vehicle injected eye). (**B**) Group of averaged ERG waveforms (a-wave amplitude, µV; b-wave amplitude, µV; a-wave latency, ms; and b-wave latency, ms). Statistical analysis between groups was performed using one-way ANOVA followed by Tukey's multiple comparisons test.

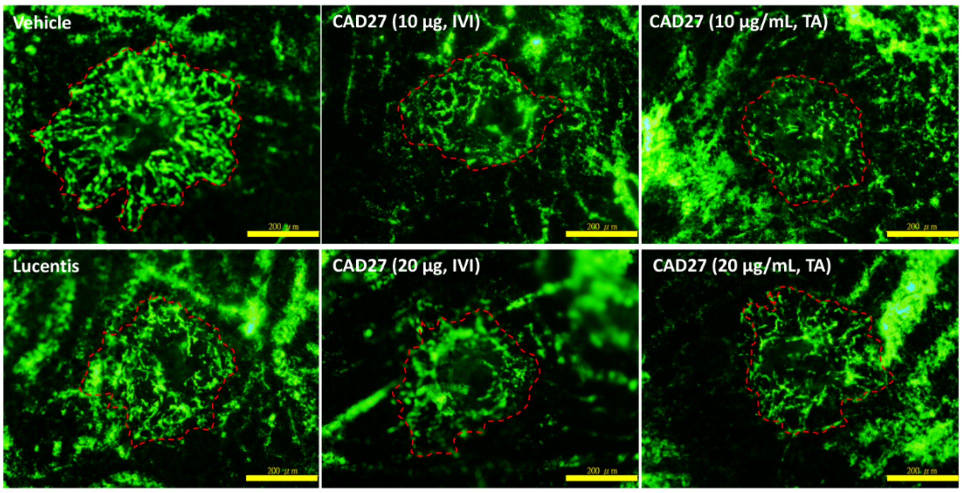

Supplementary Figure S2. The original images of the choroidal vascularity of laser-induced CNV lesions used in Figure 5A. The area with the red dashed line indicates CNV lesion. Scale bar: 200µm.

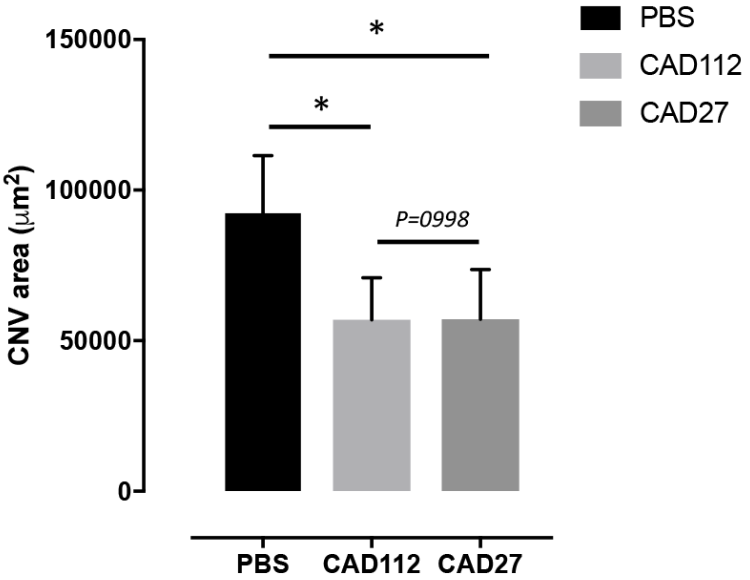

Supplementary Figure S3. Flat-mount analysis of choroidal vascularity after an intravitreal application of CAD112 and CAD27. Choroidal vascularity of laser-induced CNV lesions was examined by labeling using FITC-dextran. FITC-dextran labeling CNV in the choroidal flat-mounts was quantified and data are presented as mean ± SD (n = 6 eyes). CAD112 (10 µg/5µL), CAD27 (20 µg/5µL). using one-way ANOVA followed by Tukey's multiple comparisons test. (* $p < 0.01$).
